# Y disruption, autosomal hypomethylation and poor male lung cancer survival

**DOI:** 10.1038/s41598-021-91907-8

**Published:** 2021-06-14

**Authors:** Saffron A. G. Willis-Owen, Clara Domingo-Sabugo, Elizabeth Starren, Liming Liang, Maxim B. Freidin, Madeleine Arseneault, Youming Zhang, Shir Kiong Lu, Sanjay Popat, Eric Lim, Andrew G. Nicholson, Yasser Riazalhosseini, Mark Lathrop, William O. C. Cookson, Miriam F. Moffatt

**Affiliations:** 1 National Heart and Lung Institute, Imperial College London, London, SW3 6LY UK; 2 Department of Biostatistics, Harvard T.H. Chan School of Public Health, Boston, MA 02115 USA; 3 Program in Genetic Epidemiology and Statistical Genetics, Harvard T.H. Chan School of Public Health, Boston, MA 02115 USA; 4 Department of Twin Research and Genetic Epidemiology, School of Life Course Sciences, King’s College London, Lambeth Palace Road, London, SE1 7EH UK; 5 McGill Genome Centre, Montréal, QC H3A 0G1 Canada; 6 Royal Marsden Hospital NHS Foundation Trust, London and Surrey, UK; 7 The Institute of Cancer Research, 123 Old Brompton Road, London, SW7 3RP UK; 8 Department of Thoracic Surgery, Royal Brompton Hospital, Sydney Street, London, SW3 6NP UK; 9 Department of Histopathology, Royal Brompton and Harefield NHS Foundation Trust, London, UK; 10 Department of Human Genetics, McGill University, Montréal, QC Canada

**Keywords:** Non-small-cell lung cancer, Gene expression

## Abstract

Lung cancer is the most frequent cause of cancer death worldwide. It affects more men than women, and men generally have worse survival outcomes. We compared gene co-expression networks in affected and unaffected lung tissue from 126 consecutive patients with Stage IA–IV lung cancer undergoing surgery with curative intent. We observed marked degradation of a sex-associated transcription network in tumour tissue. This disturbance, detected in 27.7% of male tumours in the discovery dataset and 27.3% of male tumours in a further 123-sample replication dataset, was coincident with partial losses of the Y chromosome and extensive autosomal DNA hypomethylation. Central to this network was the epigenetic modifier and regulator of sexually dimorphic gene expression, *KDM5D*. After accounting for prognostic and epidemiological covariates including stage and histology, male patients with tumour *KDM5D* deficiency showed a significantly increased risk of death (Hazard Ratio [HR] 3.80, 95% CI 1.40–10.3, *P* = 0.009). *KDM5D* deficiency was confirmed as a negative prognostic indicator in a further 1100 male lung tumours (HR 1.67, 95% CI 1.4–2.0, *P* = 1.2 × 10^–10^). Our findings identify tumour deficiency of *KDM5D* as a prognostic marker and credible mechanism underlying sex disparity in lung cancer.

## Introduction

Sex differences in lifetime risk and survival are recognised across several common cancers^1^. In the UK, lung cancer incidence and mortality following age adjustment are 46% and 53% higher in males than females respectively^2^. As more women have taken up cigarette smoking, the gap between male and female lung cancer incidence rates is narrowing. Nevertheless, males continue to demonstrate an excess of cases and a relative survival disadvantage. Males with lung cancer have an increased risk of death at 5 years compared with females irrespective of stage, age, period of diagnosis and histologic type^3,4^. The mechanisms responsible for worse outcomes in males have not yet been established but appear to be independent of cigarette smoking, co-morbidities and treatment type^5^.

An abundance of gene expression changes accompanies lung cancer. The scale and diversity of these changes have made it difficult to discern central pathogenic processes and their relationship with prognosis. In the present study we therefore analysed gene expression at a system level, comparing transcriptome organisation between tumour and matched unaffected pulmonary tissue in NSCLC (non-small-cell lung cancer) patients undergoing surgical resection with curative intent without pre-operative adjuvant therapy. Through the application of Weighted Gene Co-expression Network Analysis (WGCNA)^6^ we were able to identify gene co-expression networks that were common to, or divergent between, tumour and histologically normal pulmonary tissue. These networks, in turn, were related to patient attributes including sex.

## Results

Human whole transcriptome data were generated from pulmonary tumours and, with few exceptions, matched unaffected tissue (referred to hereon as ‘normal’) using the Affymetrix HuGene 1.1 ST microarray. Following quality control, a total of 18,717 transcripts and 237 samples were available for analysis (Table 1). These samples originate from 126 patients (n 111 [T + N], 2 [N only], 13 [T only]) and were restricted to the two most frequent NSCLC subtypes: lung adenocarcinoma (LUAD) and lung squamous cell carcinoma (LUSC).

**Table 1:** Discovery and replication sample demographics.

	Normal n	Tumour n	Age µ (sd)	Sex % Male (n M/F)	Tumour stage n IA/IB/II/IIA/IIB/III/IIIA/IIIB/IV (NR)	Smoking n NS/EX/CS (NR)	Deceased n T/F (NR)
**Discovery**							
LUAD	83	92	68.46 (8.92)	49.14% (86/89)	54/37/0/22/12/0/42/0/7 (1)	23/90/58 (4)	77/95 (3)
LUSC	30	32	69.63 (6.79)	64.52% (40/22)	22/12/0/9/11/0/8/0/0 (0)	0/39/23 (0)	33/29 (0)
Overall	113	124	68.77 (8.42)	53.16% (126/111)	76/49/0/31/23/0/50/0/7 (1)	23/129/81 (4)	110/124 (3)
**Replication**							
LUAD	38	41	64.38 (8.6)	36.71% (29/50)	14/20/0/21/7/7/2/0/0 (8)	–	–
LUSC	21	23	68.2 (7.49)	75% (33/11)	5/13/2/6/6/9/1/2/0 (0)	–	–
Overall	59	64	65.75 (8.4)	50.41% (62/61)	19/33/2/27/13/16/3/2/0 (8)	–	–

Information not available is shown as –, age is expressed in years and deceased is as of the time of last follow-up.

*LUAD* lung adenocarcinoma, *LUSC* lung squamous cell carcinoma, *T* true, *F* false, *NS* never smoker, *EX* ex-smoker, *CS* current smoker, *NR* not recorded.

### Network structure

By convention, common and divergent components of transcriptome organisation are specified through the construction of consensus gene co-expression networks, derived from all samples and common across tissues (‘C’)^7^. Comparisons can then be made against networks derived separately in each tissue, allowing identification of tissue-specific networks.

We observed a strongly modular organisation amongst expressed genes, including both common (pulmonary consensus) and divergent (tumour or normal tissue-specific) co-expression networks. We found 46 networks (containing 35–881 transcript clusters [TC]) that demonstrate similar patterns of co-ordination in tumour (‘T’) and histologically normal (‘N’) lung tissue, as well as relative conservancy in their higher-order organisation (D(Preserve^*tumour, normal*^) 0.84). More than a third of transcripts (43.4%, n = 8129) however were not assigned to any consensus network, indicating relative independence or inconsistent patterns of co-ordination between tumour and histologically normal tissues.

Independent network construction in each tissue class yielded 36 networks in tumour samples (33–2220 TC) and 39 in histologically normal samples (34–4756 TC), with a relatively increased fraction of large networks defined here as containing > 1000 transcripts (C: 0% [n = 0], T: 19.4% [n = 7], N: 7.7% [n = 3]). Consistent with a hypothesis of partial tissue specificity, these single tissue analyses resulted in a markedly smaller proportion of transcripts lacking network assignment (T: 11.8% [n = 2213], N: 3.0% [n = 567]). Specifically, comparison against consensus networks defined one tumour network and five normal networks lacking a clear consensus counterpart (see Supplementary Figs. S1, S2 respectively, Fisher’s exact test − log10(*P*) ≥ 10.0).

### Sex-related tissue specificity

One network specific to histologically normal tissue (Normal: lavenderblush3) featured a highly significant relationship with biological sex (bicor 0.82 *P* = 3.72 × 10^–28^, n Obs = 113, see Supplementary Fig. S3). Modest relationships with both FEV1 (Forced Expiratory Volume in one second, bicor 0.31, *P* = 3.60 × 10^–03^, n Obs = 85) and BMI (Body Mass Index, bicor 0.23, *P* = 2.74 × 10^–02^, n Obs = 91) were also observed but did not retain significance when males and females were examined separately, indicating that these associations were mediated by sex. No significant association was seen with histology or smoke exposure (Supplementary Fig. S3). The transcripts comprising the lavenderblush3 network were significantly enriched for gonosomal (sex chromosome) inheritance (HP:0010985, *P*_adj_ 1.08 × 10^–08^) followed by histone demethylase activity (GO:0032452, *P*_adj_ 1.06 × 10^–05^). The majority of its 39 members (detailed in Supplementary Table S1) mapped to the sex chromosomes (15 to X, 16 to Y), and its autosomal members (n = 8) also showed prior evidence of sex-biased expression (e.g. *DDX43*, *NOX5, NLRP2*)^8,9^. These data indicate sex specificity in normal pulmonary gene expression, in keeping with the known impact of gonadal sex on pulmonary development and physiology.

Almost 95% of this network’s members (37/39 transcripts) lacked assignment to a consensus network, indicating near-complete divergence in co-expression patterning between tumour and histologically normal tissues. Moreover, over 41% of these transcripts (n = 16), in particular those that mapping to the Y chromosome (n = 12, 75%), lacked assignment to a tumour network indicating a specific loss rather than restructuring of co-ordination amongst Y chromosome genes in tumour tissue.

**Figure 1:**
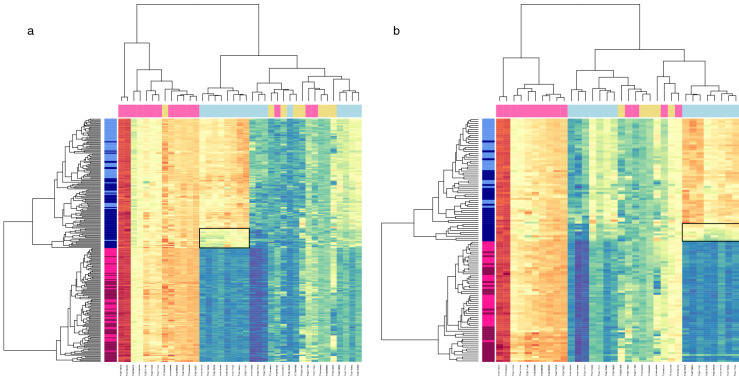
Hierarchical clustering of transcripts assigned to a normal-specific sex associated co-expression network. Figure displays heat maps with hierarchical clustering of samples (on the Y axis) and transcripts (on the X axis) in discovery (**a**) and replication (**b**) datasets, limited to transcripts clusters (TC) assigned to the normal-specific, sex associated, gene co-expression network. Expression is shown on a continuous colour scale from blue (low) to red (high). Sample colour (*y* axis) reflects tissue type (light—histologically normal, dark—tumour) and sex (blue—male, pink—female). Transcript colour (*x* axis) reflects chromosome class (yellow—autosomal, pink—X, blue—Y). Low Y sample/TCs are highlighted by a solid black box. The data presented in this Figure show broad preservation of a co-expression structure amongst these transcripts in the discovery and replication datasets and confirm the presence of a low Y chromosome expression cluster in a subset of male tumours.

The tumour-specific disturbance in sex-related gene co-expression was visualised through hierarchical clustering (Fig. 1a). The disturbance could be detected as a discrete branch characterised by a loss or substantial curtailment of male-specific gene expression, comprising more than a quarter of all male tumour samples (n = 18, 28%), including tumours of both an LUAD and LUSC histology indicating a communality of effect. Low expression across a cluster of eight Y-chromosome transcripts, as encoded by seven genes (*DDX3Y*, *EIF1AY*, *KDM5D*, *RPS4Y1*, *TXLNGY*, *USP9Y* and *UTY*), most prominently featured in the discrete branch.

Data from 34 of the 39 TC comprising the normal-specific sex-associated network were available in an independent sample of 69 lung cancer patients with LUAD or LUSC, providing 64 tumour and 59 unaffected samples (Table 1). Hierarchical clustering of these 123 samples (Fig. 1b) revealed a discrete branch bearing the hallmark of low Y-chromosome expression. The relative depression of Y chromosome expression spanned 8 transcripts, corresponding to seven Y-chromosome genes (*DDX3Y*, *EIF1AY*, *KDM5D*, *RPS4Y1*, *TXLNGY*, *USP9Y* and *UTY*); providing a complete composition match to the discovery dataset. In total the branch contained 9 male tumour samples, representing 27% of all male tumour specimens in the replication dataset.

### Loss of chromosome Y in male tumours

Mosaic loss of the Y chromosome in peripheral blood, concomitant with aging and tobacco smoke exposure^10^, is associated with increased risk for disease and mortality in men^11^ and represents a risk factor for cancer-related mortality^12^. Previous analyses of sex-chromosome aneuploidies have specified six core genes that show obligate Y chromosome dosage sensitivity in their expression^13^. Of these, all 5 available in the discovery dataset (represented on the array and meeting the described filtration criteria) were assigned to the sex-associated network in normal tissue (*TXLNGY* also known as *CYorf15B*, *DDX3Y, USP9Y*, *UTY* and *ZFY)* but lacked network assignment in either the tumour-specific or consensus datasets. This indicates a tumour-specific disruption consistent with abnormal Y chromosome dosage.

Somatic loss of Y (LOY) as a mechanism for deficiency of Y chromosome gene expression was queried in the discovery dataset through read depth analysis of whole exome sequencing (WES) and whole genome bisulfite sequencing (WGBS) data. A subset of male tumour samples exhibiting low Y expression (n WES = 6, WGBS = 17) were compared with matched unaffected tissue from the same patients and with a subset of male tumour samples lacking this feature (n WES = 9, WGBS = 7; see Supplementary Tables S3, S4) including all such samples for whom sufficient template was available. Consistent with tumour-specific LOY, normalised read depth was significantly lower in tumours exhibiting low Y-chromosome gene expression as compared with unaffected samples from the same patients (WES: two-tailed V 44718, estimate − 17.85 [95% CI − 31.58, − 4.16], *P* = 0.0108; WGBS: two-tailed V 22319, estimate − 30.34 [95% CI − 38.43, − 23.26], *P* = 2.01 × 10^–20^). This was not the case in male tumours lacking the low Y gene expression signature (WES: two-tailed V 51598, estimate − 0.08 [95% CI − 10.98, 10.83], *P* = 0.99; WGBS: two-tailed V 45987, estimate 0.06 [95% CI − 4.23, 4.34], *P* = 0.97). Correspondingly the percentage loss was significantly greater in males with low Y-expressing tumours than in males lacking this feature (WES: two-tailed t 2.499, df 13, *P* = 0.027; WGBS: two-tailed Mann–Whitney *U* 12, n_1_ 13, n_2_ 5, *P* = 0.046, see Supplementary Fig. S4a,b).

**Figure 2:**
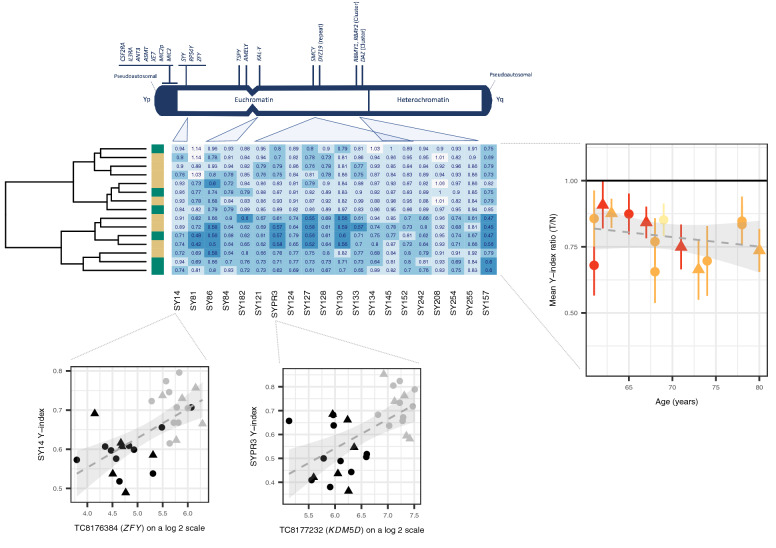
Validation of Loss of Y. Figure is headed with a cartoon adapted from the Promega technical manual depicting sites on chromosome Y interrogated through PCR. A heatmap details the ratios between tumour (T) and histologically normal (N) amplification signals on a patient-by-patient basis. Histology is shown as a y axis sidebar (LUAD = beige, LUSC = green). The grand mean and standard deviation of these ratios across all sites is plotted against age as expressed in years and coloured by smoking history (never smoker = yellow, ex-smoker = orange, current smoker = red). A hatched linear smooth line is shown with its 95% confidence intervals shaded in grey. The relationship between amplification signal (the Y-index, presented on the y axis) and the expression of genes in the same region (presented on the x axis) are shown below, with individual points coloured by tissue class (tumour = black, histologically normal = grey) and including a hatched linear smooth line with 95% confidence intervals shaded in grey. Tumour histology is denoted by point shape (LUAD = circle, LUSC = triangle).

In 16 patients with low Y expressing tumours (inclusive of the 6 assayed through WES), a polymerase chain reaction (PCR)-based chromosome deletion detection assay^14^ was used to corroborate LOY across 20 specific regions of the Y chromosome. Relative amplification of these Y-chromosome-specific loci was compared against the expression of genes located in the same physical regions confirming a positive relationship (*SYPR3*–*KDM5D* r = 0.59, df = 28, *P* = 0.0005; *SY14Y*–*ZFY* r = 0.62, df = 28, *P* = 0.0002). Matched tumour-normal data pairings were available for a total of 15 patients. The ratio between amplification indices in tumour and paired histologically normal samples was indicative of partial somatic deletion in the tumours (Fig. 2).

### Autosomal hypomethylation and LOY

Within the sex-associated gene co-expression network, network membership (MM, a metric closely related to intra-network connectivity) was highest for the gene *KDM5D* (MM 0.99, *P* = 6.21 × 10^–94^) (see Supplementary Table S1). *KDM5D* encodes a male-specific demethylase targeting trimethylated H3K4 (H3K4me3). This chromatin landmark is generally detected near the start site of transcriptionally active genes^15^ and can exhibit pronounced sex bias which translates to sex differences in gene expression^16^. In mouse embryonic fibroblasts, KDM5D-mediated H3K4 demethylation is specifically required for sex-dependent regulation of gene expression^17^. Whilst histone and DNA methylation pathways involve distinct enzymes and chemical reactions, these pathways are interconnected, with complex dependency relationships^18^. Amongst histone methylation marks H3K4me3 specifically is anti-correlated with DNA methylation^19^ and mutations in the X-linked *KDM5D* homolog (*KDM5C*) have been linked with multi-locus DNA methylation loss^20^, providing evidence of functional inter-dependency. Moreover, looking beyond the role of *KDM5D* as an epigenetic modifier, evidence accumulated from various tumour classes also points to a redistribution, or perturbation of DNA methylation upon copy number alteration^21^.

**Figure 3:**
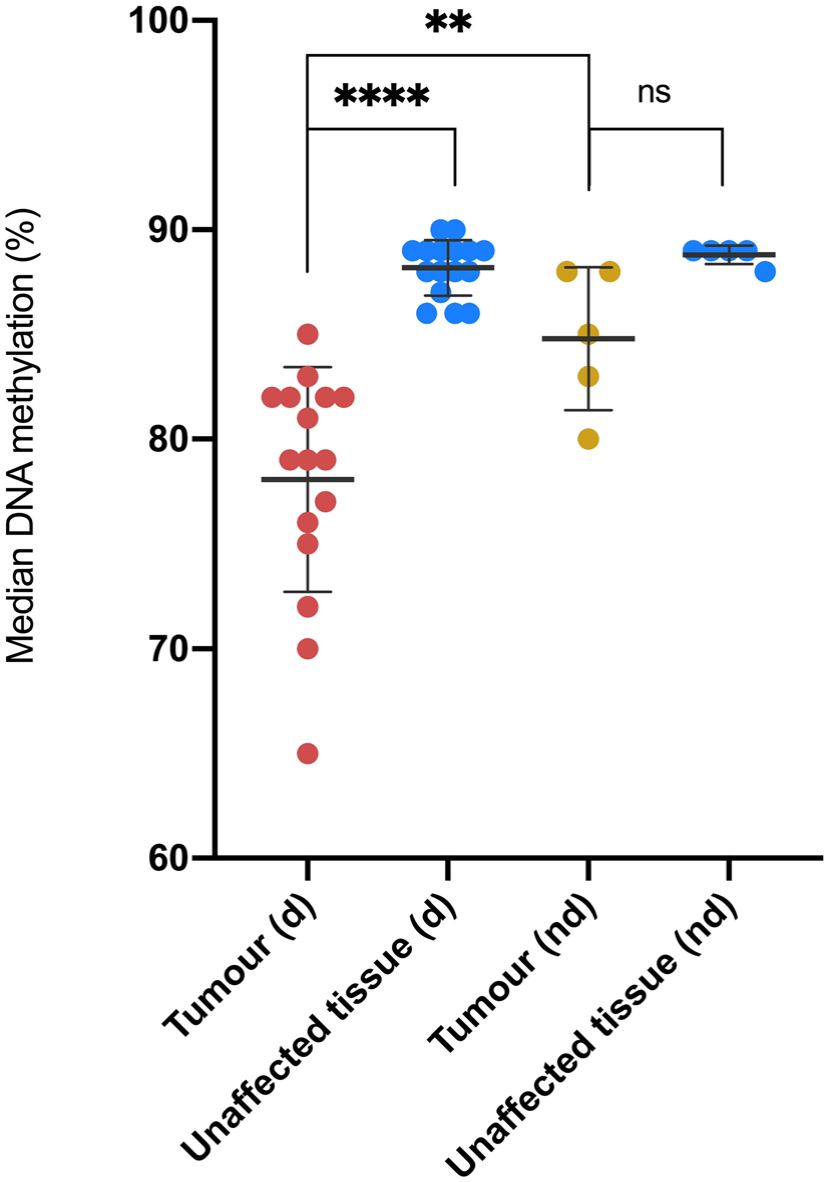
Median CpG DNA methylation percentage per sample. The figure shows median DNA CpG methylation percentage per sample in males with deficient Y chromosome gene expression (d) and males lacking this feature (nd) (see Fig. 1). Data is shown for both tumour and histologically normal tissue. Normality was assessed with a Shapiro Wilk test. Differences in DNA methylation between paired tumour and histologically normal tissues were assessed using a two-tailed paired t-test (low Y group), and a Wilcoxon test (non-low Y group). A two-tailed unpaired Mann–Whitney test was used to assess differences in DNA methylation between the two tumours groups. Error bars represent standard deviation from the mean. Magnitude of significance is denoted with asterisks (*). *d* deficient chromosome Y gene expression, *nd* non-deficient chromosome Y gene expression, *ns* non-significant.

Here we observe a pronounced DNA methylation loss signature in male tumours with the low Y gene expression phenotype (Fig. 3). Relative to paired unaffected tissues, median autosomal DNA methylation levels were significantly reduced (two-tailed t − 7.19, estimate − 10.13 [95% CI − 13.13, − 7.13], df 15, n 17, *P* = 3.12 × 10^–6^). This relative reduction was not reproduced in male tumours lacking the low Y gene expression feature (Wilcoxon matched-pairs signed rank test estimate − 3.89 [95% CI − 8.24, − 0.65], df 3, n 5, *P* = 0.0625), hence indicating that extensive hypomethylation is a specific characteristic of the low Y pulmonary tumour state and potentially therefore also a latent factor contributing to lung cancer-related methylation changes reported elsewhere^22^. Autosomal DNA methylation levels were also significantly lower in male tumours exhibiting low Y gene expression as compared with other male tumours lacking this feature (two-tailed W 8, estimate − 5.89 [95% CI − 11.41, − 1.11], n_1_ 17, n_2_ 5, *P* = 0.0082). These results demonstrate coincidence between reduced Y chromosome gene expression and widespread autosomal DNA hypomethylation in the same patients and suggest deficiency of the epigenetic modifier *KDM5D* as a potential mechanism.

Examination of individual regions showing significant differential methylation between low-Y expressing tumours and unaffected paired tissues confirmed cancer-associated changes in DNA methylation strongly biased in favour of hypomethylation. Promoter regions 1 Kb upstream of 1728 genes were found to be hypomethylated in low Y expressing tumours with methylation differences exceeding 20%. These regions showed significant enrichment for multiple motifs relating to the dimeric AP-1 (activating protein 1) transcription factor complex (see Supplementary Table S5) which has established roles in malignant transformation and invasion^23^. Hypomethylation was not, however, universal and a total of 473 promoter regions were significantly hypermethylated in low Y expressing tumours. These sites showed significant enrichment for an X-box motif, recognised by RFX transcription factors, and functioning in cellular specialization and terminal differentiation with particular relevance to ciliogenesis^24^.

### Regulation of XY dosage

*KDM5D* has a functional ancestral homolog on the X chromosome, *KDM5C*, which escapes X-inactivation and shows a male-biased pattern of deleterious mutations which associate with male cancer^25^ and DNA hypomethylation^20^. We show here that transcript abundance of *KDM5C* differs significantly between male tumours exhibiting low *KDM5D* expression (≥ 1.5 SD below the overall male mean) and male tumours lacking this feature (n 65, two-sided W = 181, difference in location − 0.21[95% CI − 0.34, − 0.06], *P* = 0.0091), with low *KDM5D* expressing tumours exhibiting relatively raised *KDM5C*. These data contrast with cardiomyocytes, where *KDM5D* knockdown has no discernible impact on *KDM5C* levels^26^, and indicate a degree of active regulation of the dosage balance between these gametologs in the lung. Moreover, these data suggest that overexpression of *KDM5C* is unable to fully compensate for deficiency of *KDM5D*.

### Prognostic value of tumour *KDM5D*

Down-regulated expression of *KDM5D* has previously been reported in the context of renal cell carcinoma^14^, prostate cancer^27^ and gastric cancer^28^; in at least a proportion of tumours due to somatic loss or segmental deletions of the Y chromosome. Clinically, low *KDM5D* expression is variably associated with a worse prognosis, more aggressive phenotype and metastasis.

At the time of last follow-up 33 male patients with tumour samples had died. Of these, 8 (24%) had markedly low male tumour *KDM5D* expression (≥ 1.5 SD below the overall male mean), meaning that almost two thirds (62%) of all males with low tumour *KDM5D* had died as opposed to 49% of males lacking this marker.

**Figure 4:**
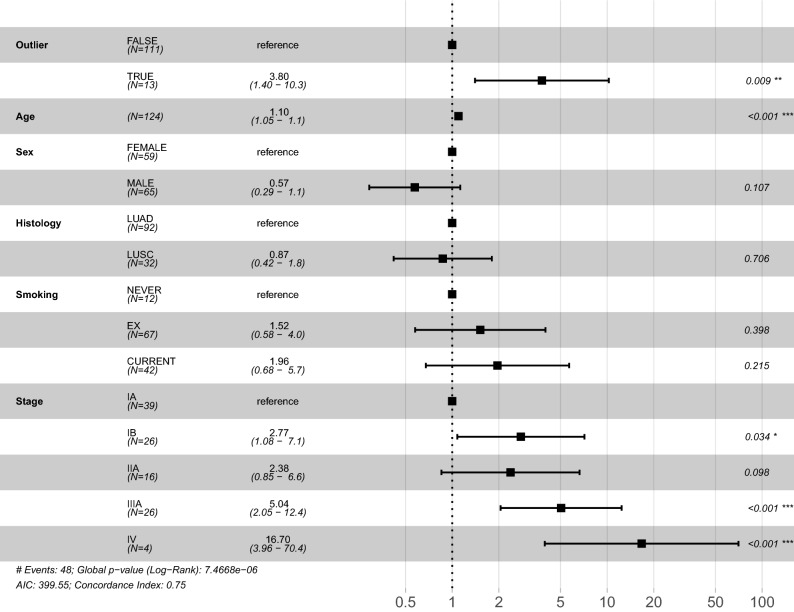
Forest plot for Cox proportional hazards model. The figure provides a forest plot reporting the hazard ratio (HR) and the 95% confidence intervals of the HR for each covariate included in the Cox proportional hazards model. The variable *Outlier* specifies male tumour samples showing relative *KDM5D* deficiency (≥ 1.5 SD below the overall male mean). Magnitude of significance is denoted with asterisks (*). *LUAD* lung adenocarcinoma, *LUSC* lung squamous cell carcinoma, *AIC* Akaike information criterion.

We sought to isolate the relationship between *KDM5D* deficiency and prognosis in lung cancer by fitting a multivariate Cox proportional hazards model in the discovery dataset (Fig. 4). Following adjustment for baseline prognostic and epidemiological covariates, including age, sex, histology, smoking history and tumour stage, markedly low tumour *KDM5D* expression in males was associated with an increased relative hazard of death as compared with females or males with normal range *KDM5D* (n 124, HR 3.80 [95% CI 1.40–10.3], *P* = 0.009). Significance was retained in an equivalent analysis restricted to males only (n 65, HR 4.92 [95% CI 1.46, 16.55], *P* = 0.01).

Notably a model evaluating the wider impact of low Y expression, as indexed by tumour membership of the low Y cluster (shown in Fig. 1a), yielded broadly similar (n 124, HR 4.20 [95% CI 1.66, 10.59], *P* = 0.002) although statistically distinguishable results (*P* = 0.0165). This indicates the presence of other prognostically relevant effects amongst the Y cluster genes.

In silico validation of the association between tumour *KDM5D* and survival was sought via the online Kaplan–Meier plotter platform (http://kmplot.com/analysis/) accessing 1100 male tumour samples derived from 11 independent lung cancer mRNA gene chip datasets. Consistent with the observations in our dataset, relatively low tumour *KDM5D* mRNA expression was associated with an unfavourable prognosis in males (HR 1.67 [95% CI 1.4, 2.0], *P* = 1.2 × 10^–10^, see Supplementary Fig. S5).

### Wider role in male predominant tumours

Tumour *KDM5D* abundance, as gauged through RNA-seq, was available through the Kaplan–Meier plotter platform across 14 non-sex-specific cancer types totalling 2423 male patients. Survival analysis incorporating low *KDM5D* as a prognostic indicator yielded nominally significant *P*-values (*P* ≤ 0.05) in seven cancers types, most significantly in head-neck squamous cell carcinoma (n 366, HR 1.79 [95% CI 1.3, 2.5], *P* = 0.0003) and liver hepatocellular carcinoma (n 249, HR 1.85 [95% CI 1.16, 2.94], *P* = 0.008). Both head and neck and liver hepatocellular carcinoma have raised incidence in males^29,30^, and within head and neck cancer male sex also carries a significant survival disadvantage. We note that the smallest *P*-values were observed in cancers where automatic thresholding placed a cut-off below 20% of the maximum recorded in that tissue (see Supplementary Table S2) suggesting a low natural split in the male abundance spectrum in some cancers. Nevertheless, when *KDM5D* abundance was alternatively split at the lowest quartile, significance was retained for both head-neck squamous cell carcinoma (HR 1.75 [95% CI 1.3, 2.5], *P* = 0.0011) and liver hepatocellular carcinoma (HR 1.82 [95% CI 1.1, 2.9], *P* = 0.0099).

## Discussion

Biological sex and sex hormone exposure have known influences on lung structure, development and physiology, and a variety of pulmonary diseases show significant sex differences in incidence, trajectory and therapeutic reponse^31^. Sex-effects on gene expression are widespread and predominantly tissue-specific^32^.

In our study we have discovered a gene co-expression network that is closely associated with sex in histologically normal lung tissue but profoundly disrupted in a subset of LUAD and LUSC tumours. We have shown that these effects are mediated by somatic LOY and co-occur with a DNA hypomethylation signature. DNA hypomethylation is a common hallmark of cancer and may contribute towards the genomic instability seen in some tumour cells^33,34^.

The male specific H3K4 demethylase KDM5D, which lies at the heart of the network, interacts with the androgen receptor in humans^35^, is required for sexually dimorphic gene expression in the mouse^17^ and may contribute towards sexual dimorphism in some immune cells^36,37^. Our observation that tumour deficiency of *KDM5D* has significant negative implications for survival is consistent with the wider deleterious effects of mosaic LOY in peripheral blood^11,12^.

*KDM5D* deletion has been recognised in 52% of prostate cancers (PC)^38^. Within this context, deficiency of *KDM5D* is associated with augmented cell cycling and accumulation of stalled replication forks, culminating in DNA-replication stress and activation of the DNA damage response kinase (ATR)^27,35^. These observations suggest the potential for interaction between *KDM5D* status and chemotherapeutic agents targeting DNA damage and repair pathways. Consistent with this hypothesis, low expression of *KDM5D* is associated with a reduced sensitivity to cisplatin and heightened sensitivity to pharmacologic inhibitors of ATR (ATRi) in PC cell lines^27^.

ATRi compounds are currently in early phase clinical trials as therapeutics or chemo-sensitizing agents^39^ with roles in replication fork stability, DNA repair and cell cycle progression. Following exposure to ATRi, *KDM5D*-deficient PC cells show curtailed proliferation and increased apoptosis indicative of a tumour-targeted synthetic lethal interaction^27^. This synergy may not be apparent in standard lung cancer cell lines such as A549 which lack evidence of Y chromosome loss^40^ (although this feature may be variably acquired through long-term culture^41^).

There is a recognised need for biomarkers capable of guiding therapeutic decision making in lung cancer. Our findings suggest that LOY-mediated curtailment of Y-chromosome gene expression, particularly deficiency of the demethylase *KDM5D*, may identify a male patient group with distinct progression and mortality profiles. It may also predict differential sensitivity to ATR pathway–targeted drugs. Moreover, given the emerging role of KDM genes^42^ including *KDM5D*^36,37^ in immune function or regulation, Y chromosome status may have implications for immunotherapy efficacy. Analyses of ATR inhibition in primary NSCLC cells under conditions of *KDM5D* knockdown and re-introduction are therefore warranted, as well as immunohistochemical studies establishing the viability of *KDM5D* detection assays.

We recognise several important limitations of our study. Samples were necessarily obtained through surgery undertaken with curative intent, resulting in an unequal representation of early- and late-stage disease. Similarly, for reasons of power, we have focused on the two most frequent histological subtypes of NSCLC which themselves have recognised genetic and epigenetic differences. Nonetheless we show here that the LOY phenomenon occurs in both LUAD and LUSC and that its impact on survival is independent of stage and histology.

Mosaic loss of the Y chromosome in circulating leukocytes constitutes the most frequent form of clonal mosaicism^43^ Therefore, without explicit quantification of immune cell content, we cannot exclude the possibility that tumour-specific LOY reflects inter-individual variation in immune cell infiltration. Nevertheless, comparison of expression levels of immune cell enriched genes, as defined in the Human Protein Atlas and available in our dataset (140 transcripts), revealed no significant evidence of augmentation in male tumours with Y chromosome disruption (data available on request), indicating that this is unlikely to be a major contributor.

Future investigations will be required to define the relative frequency of LOY by stage and histopathological subtype, as well as mapping the relationship between LOY and recognised driver mutations^43,44^, tumour immune cell content and chemotherapeutic exposures.

Here we have focussed on a single gene co-expression network that shows strong evidence of tissue specificity. Full characterisation of the remaining identified networks, and characterisation of the various mechanism(s) through which LOY impacts prognosis, remain priorities for ongoing studies.

## Methods

### Study subjects

Tumour samples and adjacent normal lung tissue were donated from surgical resections undertaken with curative intent at the Royal Brompton Hospital between 2010 and 2014, with follow-up proceeding until 2017. Written informed consent for research on biobanked tissue was obtained from all subjects. The study methodologies followed the standards set by the Declaration of Helsinki and were conducted under approval by the Royal Brompton and Harefield Research Ethics Committee (RBH) NIHR BRU Advanced Lung Disease Biobank (NRES reference 10/H0504/9) and Brompton and Harefield NHS Trust Diagnostic Tissue Bank (NRES reference 10/H0504/29) [Discovery], and the Royal Brompton and Harefield Ethics Committee (REC reference number LREC 02-261) [Replication]. Within two hours of resection tissue samples destined for transcriptomics were stored in RNAlater (Qiagen, Crawley, UK) whilst tissue samples for genomic DNA were snap-frozen and archived at -80 °C. Histology was determined through review of pathology reports and examination of haematoxylin and eosin (H&E) stained sections (A. Nicholson).

### Gene expression

#### Discovery data set

Gene expression data from the Affymetrix HuGene 1.1 ST array were available for a total of 309 samples. Of these, 6 samples from patients with tumour types individually represented by only a single patient or lacking appropriate consent for external processing were removed. Quality of the remaining expression data was assessed through arrayQualityMetrics (3.30.0) and the RLE (Relative Log Expression) and NUSE (Normalised Unscaled Standard Errors) metrics calculated within the Bioconductor package Oligo (1.38.0). These metrics highlighted 7 samples (2.3% of the input dataset) as potentially problematic and these were removed. Raw expression data for the remaining 296 samples were RMA-treated using Oligo (1.38.0) and filtered. Specifically, transcript cluster intensity was required to exceed the data set median in 1 or more sample (genefilter 1.56.0) and be designated within the Affymetrix annotation (netaffx build 36) with a cross-hybridisation potential of 1 (unique), a non-missing mRNA assignment and as part of the main design probe set category. Together these filters yielded 18,717 transcript clusters (TC). Gene annotations were collated from the netaffx build 36 and the Bioconductor package hugene11sttranscriptcluster.db (8.5.0) as assembled from public repositories. Samples derived from patients with a lung adenocarcinoma (LUAD) or lung squamous cell carcinoma (LUSC) histology were selectively retained for analysis (Table 1) giving a total of 237 samples originating from 126 patients for analysis (Table 1). Of these, 111 patients had both tumour and normal tissue data available, 2 patients had only normal tissue data available and 13 patients had only tumour tissue available.

#### Replication data set

Gene expression data from the Affymetrix HuGene 1.1 ST array were available for a total of 123 samples from 69 patients with either a LUAD or LUSC histology (Table 1). Quality control and data pre-processing were carried out as described for the discovery dataset, yielding a final data dimension of 123 samples and 17264 TC.

### Sequencing

Whole Exome Sequencing (WES) and Whole Genome Bisulfite Sequencing (WGBS) were performed at the McGill Genome Centre, Montreal, Canada. Research samples consisted of genomic DNA extracted from surgically resected, fresh-frozen human lung tumour specimens and normal paired tissue. WES sequencing libraries were prepared with the SureSelect^XT^ Target Enrichment System (Agilent SureSelect Human All Exon V4) and sequenced with Paired-End Illumina HiSeq2000 Sequencing. Non-directional Whole Genome Bisulfite Sequencing (WGBS-Seq) libraries were constructed and sequenced with paired-end Illumina HiSeq X Next Generation Sequencing. Both WES and WGBS were performed according to standard protocols.

### PCR-based detection of LOY

The Y Chromosome Deletion Detection System assay, Version 2 (Promega, WI, USA) was performed in a total of 16 patients (31 samples, 15 complete tumour-normal tissue pairs), across 20 regions of the Y chromosome as per the manufacturer’s instructions and as detailed elsewhere^14^. Briefly, the intensity value for each Y-linked amplicon was normalized to the intensity value of corresponding (non-Y) control amplicon obtained from the same sample. The average of these values across 3 replicates, the Y-index, was used to calculate a patient-specific tumour:normal ratio. Corresponding expression data were available for all but one of these samples.

### Statistical analysis

#### Gene co-expression network analysis

A consensus network analysis of tumour and normal lung expression data was performed using step-by-step unsigned WGCNA (1.51)^45^, employing a soft-thresholding power of 5 (see Supplementary Fig. S6) and scaling topological overlap matrices (TOM) for purposes of comparability (scaling parameter 0.95). Code is available at https://github.com/cooksonmoffattlab/LOY.

Adaptive branch pruning was performed using dynamicTreeCut (1.63-1), applying a minimum cluster size of 30, a maximum joining height of 0.995 and a deep split parameter of 2 (specifying the sensitivity to cluster splitting). Modules classified as too close in terms of the correlation of their module eigengenes were merged (maximum dissimilarity that qualifies modules for merging 0.25). Consensus modules were related to phenotypic traits through two-sided bi-weight mid-correlation (robustY = FALSE, maxPOutliers = 0.05 as per recommended best practice for settings that include binary or ordinal variables) and compared with modules identified in tumour or unaffected tissue alone as calculated using equivalent computational parameters. Pathway enrichment analysis was implemented in g:profiler (e96_eg43_p13_563554d, https://biit.cs.ut.ee/gprofiler/)^46^ based on unique Entrez ID annotations (as determined through hugene11sttranscriptcluster.db 8.5.0) and incorporating the tailor-made g:SCS algorithm for multiple testing correction.

### Sequence read depth analysis

Sequencing read coverage was analysed for a total of 21 samples (15 patients, described in Supplementary Table S3) through the analysis of WES data available as part of a wider study. Sequence read coverage was obtained for all chromosome Y genes using the BEDtools (2.26.0) coverage tool and normalised both by gene length and sample sequencing depth. Percentage of loss of chromosome Y was then calculated considering only the captured regions. Normality of the data was examined through Shapiro–Wilk normality tests. Paired and un-paired t-tests were performed as appropriate, to examine between-group differences, and these were plotted using GraphPad Prism (8.3.1).

### Differential methylation

Analysis of WGBS-Seq data was performed with GenPipes^47^. The standard GenPipe for methylation analysis Methyl-Seq is adapted from the Bismark pipeline. Alignment was performed with bismark (0.18.1) and bowtie2 (2.3.1) according to bismark user guide manual with default options. SAM files thus obtained per sample were sorted by chromosomic location with GATK (Genome Analysis Tool Kit) (3.7) and read alignments deemed to be PCR duplicates were removed with Picard (2.9.0). Bismark methylation extractor was used to extract methylation in CpG context. Methylkit R package (1.12.0) was used to obtain median methylation per sample and clustering based on methylation profiles.

Calling of Differentially Methylated Regions (DMRs) was performed with Dispersion Shrinkage for Sequencing data with single replicates (DSS-single)^48^ implemented in the DSS Bioconductor R package (2.34.0) which takes into account spatial correlation, read depth and biological variation between groups. DMRs were called using the criterion absolute methylation differences > 20% and *P* < 0.001.

Coordinates 1 Kb upstream hg19 Ensembl genes were downloaded from UCSC Table Browser to obtain promoter genomic regions. Proximity of DMRs to promoter regions was analysed with Bedtools’ IntersectbED^49^. Then, enriched TF binding motifs in the genomic regions of promoters were identified by employing the motif enrichment algorithm in the HOMER (4.9.1) tool^50^. CpG normalization and use of the repeat-masked sequence were the options given for finding enriched motifs in the genomic regions given.

In order to avoid any confounding influence of low chromosome Y read depth on the measurement of Y chromosome DNA methylation, the analysis was restricted to the autosomes.

### Survival analysis

Survival curves and a multivariate Cox proportional hazards model were fitted using the R package Survival (2.44-1.1). Survival curves and forest plots were drawn using survminer (0.4.3). Model comparison was achieved through an implementation of the likelihood-ratio test for Cox regression models as proposed by Fine^51^ (nonnestcox 0.0.0.9000).

### In silico validation of tumour *KDM5D* as a prognostic marker

The prognostic value of tumour *KDM5D* in male cancer was assessed via the Kaplan–Meier plotter (http://kmplot.com/analysis/); an online platform providing access to overall survival data in combination with gene chip or RNA-seq transcriptional data^52,53^.

#### Lung cancer

Arrays designated as biased through the Kaplan–Meier plotter quality control pipeline were excluded. Overall survival was available in 1100 male patients with lung cancer split across 11 independent cohorts (CaArray, GSE14814, GSE19188, GSE29013, GSE30219, GSE31210, GSE31908, GSE37745, GSE4573, GSE50081 and TCGA). *KDM5D* was accessed through the Affymetrix ID 206700_s_at (range 3–3581) with automatic thresholding (applied cut-off 515, 14.38% of maximal).

#### Pan-cancer

The wider prognostic value of tumour *KDM5D* in male cancer outside of the lung was explored via the Kaplan–Meier plotter utilising RNA-seq data available across a total of 2423 male patients and 14 cancer types, excluding sex-specific cancers and cancers individually represented by ≤ 20 samples. These included bladder carcinoma (n = 298), esophageal adenocarcinoma (n = 69), esophageal squamous cell carcinoma (n = 69), head-neck squamous cell carcinoma (n = 366), kidney renal clear cell carcinoma (n = 344), kidney renal papillary cell carcinoma (n = 211), liver hepatocellular carcinoma (n = 249), pancreatic ductal adenocarcinoma (n = 97), pheochromocytoma and paraganglioma (n = 77), rectum adenocarcinoma (n = 90), sarcoma (n = 118), stomach adenocarcinoma (n = 238), thymoma (n = 62) and thyroid carcinoma (n = 135). Automatic thresholding was applied.

## Supplementary Information

Supplementary Information.

## Data Availability

Gene expression data have been deposited in NCBI’s Gene Expression Omnibus and are accessible through GEO SuperSeries accession number GSE151103 (https://www.ncbi.nlm.nih.gov/geo/query/acc.cgi?acc=GSE151103); comprising SubSeries GSE151101 (discovery) and GSE151102 (replication). Sequence data are available upon request.

## Code availability

Network analysis code utilised in this manuscript follows the publicly available WGCNA consensus pipeline^45^. Scripts used for sequence analysis are available upon request.
