# Supplementary material for: Y disruption, autosomal hypomethylation and poor male lung cancer survival
Source: Sci Rep. 2021 Jun 14;11:12453. doi: 10.1038/s41598-021-91907-8 (PMC8203787; doi:10.1038/s41598-021-91907-8)
Supplement: Supplementary file 1 — Supplementary Information. [file 41598_2021_91907_MOESM1_ESM.docx]

**SUPPLEMENTARY INFORMATION**

**Y disruption, autosomal hypomethylation and poor male lung cancer survival**

**Authors**

Saffron A.G. Willis-Owen^1^, Clara Domingo Sabugo^1#^, Elizabeth Starren ^1#^, Liming Liang^2,3^, Maxim B. Freidin^1,4^, Madeleine Arseneault^5^, Youming Zhang^1^, Shir Kiong Lu^1^, Sanjay Popat^6,7^, Eric Lim^8^, Andrew G. Nicholson^1,9^, Yasser Riazalhosseini^5,10^, Mark Lathrop^5^, William O.C. Cookson^1*^, Miriam F. Moffatt^1*^.

**Affiliations**

^1^National Heart and Lung Institute, Imperial College, London SW3 6LY, UK.

^2^Department of Biostatistics, Harvard T.H. Chan School of Public Health, Boston, MA, 02115, USA.

^3^Program in Genetic Epidemiology and Statistical Genetics, Harvard T.H. Chan School of Public Health, Boston, MA, 02115, USA.

^4^Department of Twin Research and Genetic Epidemiology, School of Live Course Sciences, King's College London, Lambeth Palace Road, London, SE1 7EH, UK.

^5^McGill Genome Centre, Montréal, Quebec, H3A 0G1, Canada.

^6^Royal Marsden Hospital NHS Foundation Trust, London and Surrey, UK.

^7^The Institute of Cancer Research, 123 Old Brompton Road, London, SW7 3RP, UK.

^8^Department of Thoracic Surgery, Royal Brompton Hospital, Sydney Street, London SW3 6NP, UK.

^9^Department of Histopathology, Royal Brompton and Harefield NHS Foundation Trust, London, UK.

^10^Department Of Human Genetics, McGill University, Montréal, Quebec, Canada.

^#^These authors contributed equally to this work.

^*^Corresponding authors, these authors contributed equally to this work.

**Supplementary Table S1: A sex-associated normal-specific gene co-expression network (Normal: lavenderblush3).**

| **Transcript Cluster** | **Gene Title** | **Gene Symbol** | **Cytoband** | | **XY gametolog pair** | **XCI escape** | **MM** | **P(MM)** | **Consensus network assignment** | **Tumour network assignment** |
| --- | --- | --- | --- | --- | --- | --- | --- | --- | --- | --- |
| 8177232 | Lysine (K)-specific demethylase 5D | *KDM5D* | | Yq11.222 | *KDM5C/KDM5D* |  | 0.99 | 6.21E-94 | N | N |
| 8177137 | Ubiquitously transcribed tetratricopeptide repeat containing, Y-linked | *UTY* | | Yq11.221 | *KDM6A/UTY* |  | 0.99 | 2.40E-90 | N | N |
| 8176375 | Ribosomal protein S4, Y-linked 1 | *RPS4Y1* | | Yp11.31 | *RPS4X/RPS4Y1* |  | 0.99 | 7.97E-90 | N | N |
| 8176578 | Ubiquitin specific peptidase 9, Y-linked | *USP9Y* | | Yq11.21 | *USP9X/USP9Y* |  | 0.99 | 1.59E-89 | N | N |
| 8176624 | DEAD (Asp-Glu-Ala-Asp) box helicase 3, Y-linked | *DDX3Y* | | Yq11.21 | *DDX3X/DDX3Y* |  | 0.98 | 3.93E-84 | N | N |
| 8176698 | Taxilin gamma pseudogene, Y-linked | *TXLNGY* | | Yq11.222 | *TXLNG/TXLNGY* |  | 0.98 | 5.36E-84 | N | N |
| 8176709 | Taxilin gamma pseudogene, Y-linked | *TXLNGY* | | Yq11.222 | *TXLNG/TXLNGY* |  | 0.98 | 1.93E-75 | N | N |
| 8176719 | Eukaryotic translation initiation factor 1A, Y-linked | *EIF1AY* | | Yq11.223 | *EIF1AX/EIF1AY* |  | 0.97 | 2.53E-73 | N | N |
| 8176384 | Zinc finger protein, Y-linked | *ZFY* | | Yp11.31 | *ZFX/ZFY* |  | 0.97 | 1.25E-72 | N | N |
| 8176460 | Protein kinase, Y-linked, pseudogene | *PRKY* | | Yp11.2 | *PRKX/PRKY* |  | 0.96 | 2.93E-63 | N | Y |
| 8177214 | Testis-specific transcript, Y-linked 14 (non-protein coding) | *TTTY14* | | Yq11.222 |  |  | 0.94 | 4.37E-55 | N | N |
| 8177217 | Testis-specific transcript, Y-linked 14 (non-protein coding) | *TTTY14* | | Yq11.222 |  |  | 0.93 | 2.75E-51 | N | N |
| 8177261 | Testis-specific transcript, Y-linked 10 (non-protein coding) | *TTTY10* | | Yq11.223 |  |  | 0.92 | 1.52E-47 | N | Y |
| 8176469 | --- | *---* | | Yp11.2 |  |  | 0.92 | 1.08E-45 | N | N |
| 8176655 | Neuroligin 4, Y-linked | *NLGN4Y* | | Yq11.221 | *NLGN4X/NLGN4Y* |  | 0.91 | 5.19E-44 | N | Y |
| 8166956 | Lysine (K)-specific demethylase 6A | *KDM6A* | | Xp11.3 | *KDM6A/UTY* | Y | -0.84 | 1.11E-30 | N | Y |
| 8172827 | Lysine (K)-specific demethylase 5C | *KDM5C* | | Xp11.22 | *KDM5C/KDM5D* | Y | -0.82 | 1.25E-28 | N | Y |
| 8166500 | Zinc finger protein, X-linked | *ZFX* | | Xp22.11 | *ZFX/ZFY* | Y | -0.78 | 1.38E-24 | N | Y |
| 8171229 | Patatin-like phospholipase domain containing 4 | *PNPLA4* | | Xp22.31 |  | Y | -0.77 | 1.20E-23 | N | Y |
| 8173513 | Ribosomal protein S4, X-linked | *RPS4X* | | Xq13.1 | *RPS4X/RPS4Y1; RPS4X/RPS4Y2* | Y | -0.76 | 8.04E-23 | N | Y |
| 8171222 | Pseudouridine 5-phosphatase | *PUDP* | | Xp22.31 |  | Y | -0.75 | 5.84E-22 | N | Y |
| 8168412 | JPX transcript, XIST activator (non-protein coding) | *JPX* | | Xq13.2 |  | Y | -0.72 | 3.74E-19 | N | Y |
| 8172876 | Structural maintenance of chromosomes 1A | *SMC1A* | | Xp11.22 |  | Y | -0.67 | 4.34E-16 | Y | Y |
| 8165866 | Steroid sulfatase (microsomal), isozyme S | *STS* | | Xp22.31 |  | Y | -0.67 | 4.75E-16 | N | Y |
| 8171148 | Arylsulfatase D | *ARSD* | | Xp22.33 |  | Y | -0.65 | 3.80E-15 | Y | Y |
| 8166493 | Eukaryotic translation initiation factor 2, subunit 3 gamma, 52kDa | *EIF2S3* | | Xp22.11 |  | Y | -0.57 | 5.50E-11 | N | Y |
| 8176442 | Transducin (beta)-like 1, Y-linked | *TBL1Y* | | Yp11.2 | *TBL1X/TBL1Y* |  | 0.53 | 1.37E-09 | N | Y |
| 8120679 | DEAD (Asp-Glu-Ala-Asp) box polypeptide 43 | *DDX43* | | 6q13 |  |  | 0.50 | 1.61E-08 | N | N |
| 8166195 | Zinc finger (CCCH type), RNA binding motif and serine/arginine rich 2 | *ZRSR2* | | Xp22.2 |  | Y | -0.49 | 3.07E-08 | N | Y |
| 7984488 | Sperm equatorial segment protein 1 /// NADPH oxidase, EF-hand calcium binding domain 5 | *SPESP1 /// NOX5* | | 15q23 |  |  | 0.41 | 7.12E-06 | N | N |
| 8166182 | --- | *---* | | Xp22.2 |  |  | -0.40 | 9.32E-06 | N | Y |
| 8036862 | Uncharacterized LOC100507646 | *LOC100507646* | | 19q13.2 |  |  | -0.39 | 1.66E-05 | N | Y |
| 8171760 | Small Cajal body-specific RNA 9-like | *SCARNA9L* | | Xp22.12 |  | Y | -0.38 | 2.71E-05 | N | Y |
| 8171624 | Adhesion G protein-coupled receptor G2 | *ADGRG2* | | Xp22.13 |  |  | -0.35 | 1.32E-04 | N | Y |
| 7975694 | --- | *---* | | 14q24.3 |  |  | 0.32 | 4.78E-04 | N | N |
| 8031398 | NLR family, pyrin domain containing 2 | *NLRP2* | | 19q13.42 |  |  | -0.32 | 5.02E-04 | N | Y |
| 8153835 | Uncharacterized LOC101928953 /// Uncharacterized LOC105375802 | *LOC101928953 /// LOC105375802* | | 8q24.3 |  |  | -0.29 | 1.81E-03 | N | Y |
| 8044605 | PAX8 antisense RNA 1 | *PAX8-AS1* | | 2q13 |  |  | 0.25 | 8.75E-03 | N | N |
| 7943158 | Small Cajal body-specific RNA 9 | *SCARNA9* | | 11q21 |  |  | -0.02 | 8.01E-01 | N | Y |

Abbreviations: XCI (x chromosome inactivation); Y (yes); N (no); MM (membership metric).

N.B. XCI escape genes are listed as defined in^54,55^. XY gametolog pairs are listed as defined in^56,57^.

**Supplementary Table S2: Survival analysis for *KDM5D* abundance in non-sex-specific cancers outside the lung, as implemented in the Kaplan-Meier plotter using RNA-seq data.**

| **Cancer type** | **n Males** | **HR** | **P** | **Cut-off** | **Range** | **Cut-off as a proportion of Max** |
| --- | --- | --- | --- | --- | --- | --- |
| **Head-neck squamous cell carcinoma** | **366** | **0.56 (0.4-0.77)** | **3.40E-04** | **334** | **1-3165** | **10.55** |
| **Liver hepatocellular carcinoma** | **249** | **0.54 (0.34-0.86)** | **8.10E-03** | **237** | **13-2756** | **8.60** |
| Rectum adenocarcinoma | 90 | 0.25 (0.08-0.79) | 1.05E-02 | 327 | 34-2118 | 15.44 |
| Kidney renal papillary cell carcinoma | 211 | 2.4 (1.16-4.94) | 1.40E-02 | 379 | 0-2935 | 12.91 |
| Esophageal adenocarcinoma | 69 | 0.45 (0.22-0.91) | 2.25E-02 | 400 | 30-4717 | 8.48 |
| Bladder carcinoma | 298 | 0.67 (0.47-0.95) | 2.48E-02 | 795 | 6-5858 | 13.57 |
| Pheochromocytoma and paraganglioma | 77 | 7.25 (0.75-69.85) | 4.50E-02 | 1322 | 80-2703 | 48.91 |
| Pancreatic ductal adenocarcinoma | 97 | 0.54 (0.29-1.02) | 5.43E-02 | 1020 | 11-2953 | 34.54 |
| Kidney renal clear cell carcinoma | 344 | 1.44 (0.98-2.1) | 5.88E-02 | 853 | 0-3541 | 24.09 |
| Thymoma | 62 | 0 (0-Inf) | 8.60E-02 | 2275 | 213-4827 | 47.13 |
| Stomach adenocarcinoma | 238 | 1.37 (0.9-2.08) | 1.42E-01 | 918 | 30-4159 | 22.07 |
| Esophageal squamous cell carcinoma | 69 | 0.54 (0.2-1.46) | 2.18E-01 | 744 | 47-2738 | 27.17 |
| Sarcoma | 118 | 0.71 (0.36-1.39) | 3.17E-01 | 1159 | 30-5573 | 20.80 |
| Thyroid carcinoma | 135 | 2.55 (0.29-22.38) | 3.83E-01 | 1173 | 0-5289 | 22.18 |
| **Sum** | 2423 |  |  |  |  |  |

Table S2 details results from the Kaplan-Meier plotter (<http://kmplot.com/analysis/>) based on RNA-seq data and *KDM5D* abundance segregated into high and low bins via automatic thresholding (cut-off and range supplied). The generated HR relates to high target expression.

Abbreviations: HR (hazard ratio).

**Supplementary Table S3: Dataset demographics for sequence read depth analysis.**

|  | **n Males** | **Histology n**  **LUAD/LUSC (NR)** | **Age**  **µ(sd)** | **Tumour stage n**  **IA/IB/II/IIA/IIB/**  **III/IIIA/IIIB/IV (NR)** | **Smoking n**  **NS/EX/CS (NR)** | **Deceased n T/F (NR)** |
| --- | --- | --- | --- | --- | --- | --- |
| Low Y expression | 6 | 2/4 (0) | 66 (3.35) | 0/2/0/2/1/0/1/0/0 (0) | 0/2/4 (0) | 4/2 (0) |
| Non-low Y expression | 9 | 5/4 (0) | 67.67 (8.70) | 1/1/0/2/2/0/3/0/0 (0) | 0/6/3 (0) | 3/6 (0) |
| Overall | 15 | 7/8 (0) | 67 (6.93) | 1/3/0/4/3/0/4/0/0 (0) | 0/8/7 (0) | 7/8 (0) |

Abbreviations: LUAD (lung adenocarcinoma); LUSC (lung squamous cell carcinoma); NS (non-smoker); EX (ex-smoker); CR (current smoker); NR (not recorded); T (true); F (false); sd (standard deviation).

**Supplementary Table S4: Dataset demographics for methylation analysis.**

|  | **n Males** | **Histology n**  **LUAD/LUSC (NR)** | **Age**  **µ (sd)** | **Tumour stage n**  **IA/IB/II/IIA/IIB/**  **III/IIIA/IIIB/IV (NR)** | **Smoking n**  **NS/EX/CS (NR)** | **Deceased n T/F (NR)** |
| --- | --- | --- | --- | --- | --- | --- |
| Low Y expression | 17 | 11/6 (0) | 69.29 (6.43) | 5/3/0/3/1/0/4/0/1 (0) | 1/10/6 (0) | 10/7 (0) |
| Non-low Y expression | 5 | 5/0 (0) | 71.20 (8.35) | 2/1/0/0/0/0/1/0/1 (0) | 0/3/2 (0) | 1/4 (0) |
| Overall | 22 | 16/6 (0) | 69.73 (6.74) | 7/4/0/3/1/0/5/0/2 (0) | 1/13/8 (0) | 11/11 (0) |

Abbreviations: LUAD (lung adenocarcinoma); LUSC (lung squamous cell carcinoma); NS (non-smoker); EX (ex-smoker); CR (current smoker); NR (not recorded); T (true); F (false); sd (standard deviation).

**Supplementary Table S5: HOMER known motifs enriched in hypomethylated (a) and hypermethylated (b) promoter regions.**

**
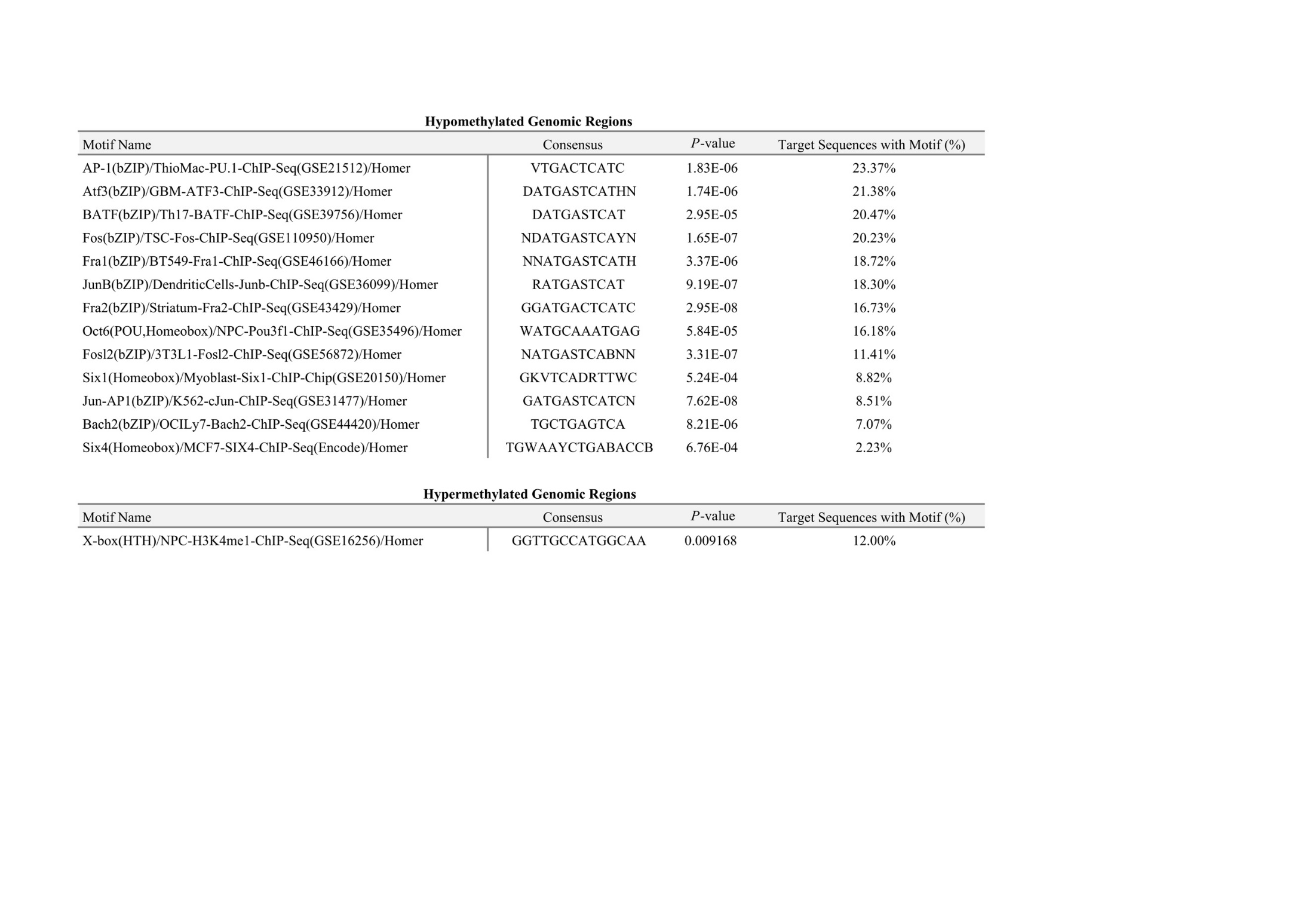
**

**a**

**b**

Motif names are designated by the transcription factor name and its DNA binding domain, followed by the GEO Accession number for the public Chromatin Immunoprecipitation Sequencing (ChIP-Seq) experiment from the Genomic Spatial Event (GSE) database. Abbreviations: basic Leucine Zipper Domain (bZIP); POU, Homeobox (POU-domain homeobox transcription factor).

**Supplementary Figures**

**Supplementary Figure 1: Correspondence of Tumour set−specific and consensus*^tumour, normal^* modules.**

Each row represents a tumour network and each column represents a consensus^tumour,normal^ network. The correspondence between each combination of networks is presented as the number of overlapping transcript clusters and the significance (-log10(*P*)) of overlap, as derived from a Fisher’s Exact Test, presented as a colour scale. Tumour networks showing high levels of overlap (-log10(*P*) ≥ 10.0) with the consensus grey designation (representing unassigned transcripts) are denoted with *. Abbreviations: Cons (Consensus).

**Supplementary Figure 2: Correspondence of Normal set−specific and consensus*^tumour, normal^* modules.**

Each row represents a normal network and each column represents a consensus^tumour,normal^ network. The correspondence between each combination of networks is presented as the number of overlapping transcript clusters and the significance (-log10(*P*)) of overlap, as derived from a Fisher’s Exact Test, presented as a colour scale. Normal networks showing high levels of overlap (-log10(*P*) ≥ 10.0) with the consensus grey designation (representing unassigned transcripts) are denoted with *. Abbreviations: Cons (Consensus).

**Supplementary Figure 3: Normal-specific network−trait relationships.**

Each row represents a network detected in histologically normal samples. Each column represents a measured trait. Figures show the network-trait biweight mid-correlation and its associated *P*-value beneath. The magnitude and direction of correlation is additionally indicated through a blue-white-red colour scale. Abbreviations: PYH (pack year history), BMI (body mass index), FEV1 (forced expiratory volume in 1 second), LUAD (lung adenocarcinoma), LUSC (lung squamous cell carcinoma).

**Supplementary Figure 4: Validation of somatic LOY through sequence read depth analysis from WES (a) and WGBS (b) data.**

**
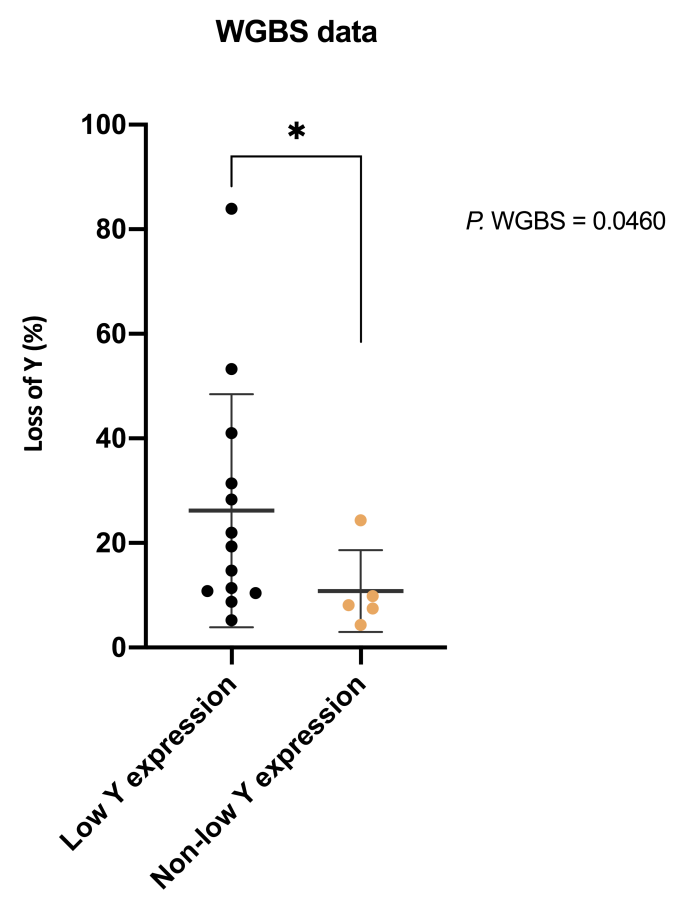

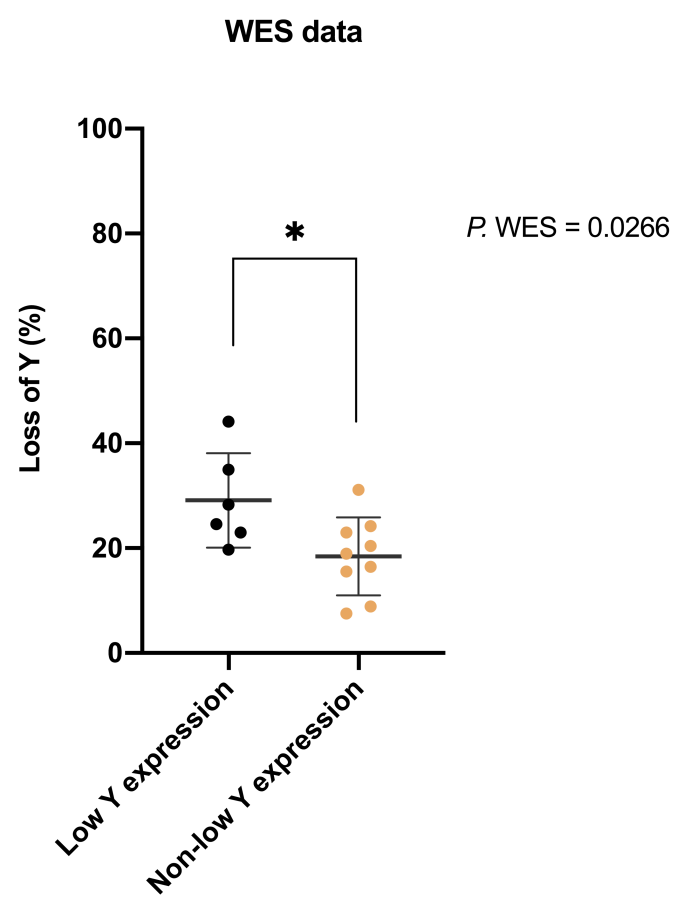
a b**

Validation of somatic LOY through sequence read depth analysis from WES (a) and WGBS (b) data. Extended data figures 4 a and b show percentage of Y loss in male tumours with low Y-expression and in males lacking this feature. The distribution of read depth sequencing data was assessed for normality using a Shapiro Wilk test with 95% confidence interval. Two-tailed unpaired t-tests and Mann-Whitney tests were applied accordingly. Error bars represent standard deviations from the mean. Magnitude of significance is denoted with asterisks (*). Abbreviations: WES (whole exome sequencing), WGBS (whole genome bisulfite sequencing).

**Supplementary Figure 5: Kaplan–Meier survival curves for low *KDM5D* in 1,100 male lung cancer samples.**

Kaplan–Meier survival curves for low *KDM5D,* as indexed by Affymetrix ID 206700_s_at, in 1,100 male lung cancer samples calculated using the online Kaplan-Meier plotter platform (<http://kmplot.com/analysis/>)^58^. Probability of survival (y axis) is shown as a function of time in months (x axis). Abbreviations: HR (hazard ratio).

**Supplementary Figure 6: WGCNA summary network indices.**

Network summary statistics including scale free topology model fit, and three indices of connectivity are shown here (*y* axis) as a function of soft thresholding power (*x* axis), as varied from 1 to 20.

**References**

54 Dunford, A. *et al.* Tumor-suppressor genes that escape from X-inactivation contribute to cancer sex bias. *Nat Genet* **49**, 10-16, doi:10.1038/ng.3726 (2017).

55 Jansen, R. *et al.* Sex differences in the human peripheral blood transcriptome. *BMC Genomics* **15**, 33, doi:10.1186/1471-2164-15-33 (2014).

56 Bellott, D. W. *et al.* Mammalian Y chromosomes retain widely expressed dosage-sensitive regulators. *Nature* **508**, 494-499, doi:10.1038/nature13206 (2014).

57 Skaletsky, H. *et al.* The male-specific region of the human Y chromosome is a mosaic of discrete sequence classes. *Nature* **423**, 825-837, doi:10.1038/nature01722 (2003).

58 Gyorffy, B., Surowiak, P., Budczies, J. & Lanczky, A. Online survival analysis software to assess the prognostic value of biomarkers using transcriptomic data in non-small-cell lung cancer. *PLoS One* **8**, e82241, doi:10.1371/journal.pone.0082241 (2013).
